# LncRNA miR143HG调控肺鳞癌H520细胞生物学行为的机制研究

**DOI:** 10.3779/j.issn.1009-3419.2023.106.21

**Published:** 2023-10-20

**Authors:** Longfei GOU, Yayuan HE, Pengcheng QIU, Bo HUANG

**Affiliations:** ^1^121001 锦州，锦州医科大学; ^1^Jinzhou Medical University, Jinzhou 121001, China; ^2^121012 锦州，锦州医科大学附属第一医院胸外科; ^2^Department of Thoracic Surgery, The First Affiliated Hospital of Jinzhou Medical University, Jinzhou 121012, China

**Keywords:** LncRNA miR143HG, miR-155, 肺鳞癌, 细胞增殖, 细胞迁移, LncRNA miR143HG, miR-155, Lung squamous cell carcinoma, Cell proliferation, Cell migration

## Abstract

**背景与目的:**

肺鳞癌（lung squamous cell carcinoma, LUSC）具有高发病率和高致死率，并且其临床预后较差。然而，目前对于LUSC尚无有效的靶向治疗方案。LncRNA miR143HG作为一种长链非编码RNA（long non- coding RNA, lncRNA），已被证实在多种肿瘤的发生发展中发挥着重要作用。然而lncRNA miR143HG在LUSC细胞中所扮演的生物学角色尚不清楚。因此，本研究旨在探讨lncRNA miR143HG调控LUSC H520细胞生物学行为的作用机制。

**方法:**

基于肿瘤基因组图谱（The Cancer Genome Atlas, TCGA）数据库，对lncRNA miR143HG进行泛癌分析和差异表达分析。使用受试者工作特征（receiver operating characteristic, ROC）曲线和timeROC曲线评估lncRNA miR143HG对LUSC诊断和预后的预测效果。确定各通路对于lncRNA miR143HG的富集程度。利用实时定量聚合酶链反应（quantitative real-time polymerase chain reaction, qRT-PCR）检测BEAS-2B和H520细胞中lncRNA miR143HG以及miR-155的表达。将H520细胞随机分为空白对照组（不做任何处理）、阴性对照组（转染lncRNA-negative control，即lncRNA-NC）、lncRNA miR143HG组（转染lncRNA miR143HG）和lncRNA miR143HG+miR-155组（共转染lncRNA miR143HG和miR-155）。采用CCK-8法检测细胞增殖能力；划痕实验检测细胞迁移能力；Transwell实验检测细胞侵袭能力；流式细胞术检测细胞凋亡率；qRT-PCR和蛋白免疫印迹实验检测Wnt/β-Catenin通路相关基因和蛋白的表达情况。

**结果:**

泛癌分析和差异分析结果共同显示，除肾透明细胞癌外，其他癌组织中lncRNA miR143HG的表达均高于健康组织，且在LUSC中的差异具有统计学意义。ROC曲线和timeROC曲线的评估结果提示lncRNA miR143HG对于LUSC的诊断及预后的预测具有重要意义。富集于lncRNA miR143HG高表达的通路主要包括黏着斑、血管平滑肌收缩和钙信号通路等；而富集在lncRNA miR143HG低表达的通路主要包括氧化磷酸化、细胞周期、基础转录因子等。qRT-PCR结果显示，与BEAS-2B细胞相比，lncRNA miR143HG在H520细胞中低表达，而miR-155在H520细胞中高表达（P<0.05）。与阴性对照组比较，lncRNA miR143HG组细胞中lncRNA miR143HG基因、Wnt的基因和蛋白以及β-Catenin的基因和蛋白表达水平显著增加，而miR-155基因表达显著降低，且细胞增殖能力、细胞迁移能力和细胞侵袭能力也明显降低，但细胞凋亡率明显升高（P<0.05）。另外，与lncRNA miR143HG组比较，过表达miR-155能够逆转lncRNA miR143HG介导的生物学行为，差异具有统计学意义（P<0.05）。

**结论:**

LncRNA miR143HG对于H520细胞的生物学行为具有重要意义。LncRNA miR143HG通过下调miR-155表达抑制H520细胞的增殖、迁移和侵袭能力并促进H520细胞的凋亡，其分子机制可能与Wnt/β-Catenin通路相关。

## Abstract (en)

**Background and objective:**

There is a high morbidity, mortality, and poor clinical prognosis of lung squamous cell carcinoma (LUSC). However, there is currently no effective targeted treatment plan for LUSC. As a long non-coding RNA (lncRNA), lncRNA miR143HG has been proven to play an important role in the occurrence and development of various tumors. However, the biological role played by lncRNA miR143HG in LUSC cells is still unclear. Therefore, this study aimed to investigate the mechanism of lncRNA miR143HG on regulating the biological behavior of LUSC H520 cells.

**Methods:**

Pan-cancer analysis and differential expression analysis of lncRNA miR143HG were performed based on The Cancer Genome Atlas (TCGA) database. The predictive effect of lncRNA miR143HG on the diagnosis and prognosis of LUSC was evaluated by adopting the receiver operating characteristic (ROC) curve and timeROC curve. The enrichment degree of each pathway to lncRNA miR143HG was determined. The expression of lncRNA miR143HG and miR-155 in BEAS-2B cells and H520 cells was detected using quantitative real-time polymerase chain reaction (qRT-PCR). H520 cells were randomly divided into blank control group (without any treatment), negative control group (transfected with lncRNA-NC), lncRNA miR143HG group (transfected with lncRNA miR143HG), and lncRNA miR143HG+miR-155 group (co-transfected with lncRNA miR143HG and miR-155). The approaches of CCK-8, wound healing test, Transwell assay, flow cytometry, qRT-PCR, and Western blot were respectively employed to detect the cell proliferation ability, cell migration ability, cell invasion ability, cell apoptosis rate, and expression level of related genes and proteins of the Wnt/β-Catenin pathway.

**Results:**

The results of pan-cancer analysis and differential analysis collectively showed that except for renal clear cell carcinoma, the expression of lncRNA miR143HG in other cancer tissues was higher than that in healthy tissues, and the differences were significant in LUSC. The evaluation results of the ROC curve and timeROC curve suggested that lncRNA miR143HG was of great significance in the prediction of diagnosis and prognosis of LUSC. The pathways enriched in high expression of lncRNA miR143HG mainly included focal adhesion, vascular smooth muscle contraction, calcium signaling pathways, and so on; the pathways enriched in the low expression of lncRNA miR143HG embraced oxidative phosphorylation, cell cycle, basic transcription factors, etc. The qRT-PCR results showed that lncRNA miR143HG was low expressed but miR-155 was highly expressed in H520 cells when compared to BEAS-2B cells (P<0.05). Compared with the negative control group, the expression levels of the gene of lncRNA miR143HG, the gene and protein of Wnt, as well as the gene and protein of β-Catenin were significantly increased, while the gene expression of miR-155, the ability of cell proliferation, cell migration, and cell invasion were significantly reduced, but the cell apoptosis rate was dominantly elevated in cells of lncRNA miR143HG group (P<0.05). In addition, compared with the lncRNA miR143HG group, overexpression of miR-155 could reverse the biological behavior mediated by lncRNA miR143HG, and the difference was statistically significant (P<0.05).

**Conclusion:**

LncRNA miR143HG was of great significance for the biological behavior of H520 cells. LncRNA miR143HG inhibited the ability of proliferation, migration, and invasion, as well as enhanced the apoptosis of H520 cells by downregulating miR-155 expression, which may be related to the Wnt/β-Catenin pathway.

迄今为止，肺癌是最难治疗的肿瘤之一^[1]^。约85%的肺癌为非小细胞肺癌^[2]^，其中肺鳞癌（lung squamous cell carcinoma, LUSC）占肺癌的40%-51%^[3]^。LUSC每年导致全球约40万患者死亡^[4]^。临床上，放疗和化疗仍是LUSC的一线治疗策略^[5]^。近年来，随着分子研究和药物研发的进步，LUSC的治疗也取得了很大进展。然而LUSC的临床预后较差，目前尚无有效的靶向治疗方案。因此深入探索针对LUSC治疗的新靶点具有重要的临床意义。

长链非编码RNA（long non-coding RNA, lncRNA）是指长度大于200个核苷酸而不具有蛋白质编码能力的RNA转录本^[6]^。LncRNAs在细胞发育和人类疾病（如癌症）中是至关重要的调节因子，能够通过不同的分子机制影响哺乳动物细胞的生物学过程，如ceRNAs、基因转录调节和表观遗传调节等^[7]^。研究^[8⇓⇓-11]^表明lncRNAs在多种肿瘤中具有潜在的致癌或抑癌活性，如肝癌、肺癌和乳腺癌等。已有证据^[12⇓-14]^显示，lncRNA miR143HG作为抑癌基因在肿瘤的发生发展中发挥着重要作用，如lncRNA miR143HG能通过靶向miR-21抑制喉鳞状细胞癌的侵袭和迁移^[15]^。此外Wang等^[16]^提出，lncRNA miR143HG通过特异性吸附miR-504能够抑制胶质母细胞瘤细胞增殖。然而lncRNA miR143HG在LUSC细胞中所扮演的生物学角色尚不清楚。

微小RNA（microRNAs, miRNAs）已逐渐成为多种癌症发生和发展的关键分子，显示出调节关键致癌途径的重要能力^[17]^。作为最保守的多功能miRNAs之一，miR-155表达的变化与各种生理和病理过程有关，包括免疫反应、炎症和肿瘤发生^[18]^。多项研究^[19⇓-21]^表明，miR-155的过度表达是导致多种恶性肿瘤增殖、迁移、侵袭等多种生物学行为的重要因素之一。尽管miR-155作为一种致癌基因以促进LUSC的发生发展已被证实，然而在LUSC的发生发展过程中，miR-155是如何被过度表达的相关机制尚未完全明确，尤其是受lncRNA miR143HG调节的研究相对较少。此外，相关研究^[22]^表明Wnt/β-Catenin信号通路的异常调节与恶性肿瘤的进展、肿瘤的不良预后以及癌症致死率等密切相关。有研究^[23]^提出有效调节Wnt/β-Catenin信号通路可能为各种类型的癌症提供有希望的治疗选择，包括LUSC的治疗。然而，在调节lncRNA miR143HG和miR-155基因后，Wnt/β-Catenin信号通路是否会被进一步调节的相关研究鲜有报道。因此在本研究中，我们基于miR-155基因和Wnt/β-Catenin信号通路，重点探讨了lncRNA miR143HG对LUSC细胞生物学行为的影响以及潜在的分子机制。

## 1 资料与方法

### 1.1 主要试剂与仪器

正常肺上皮细胞BEAS-2B和LUSC细胞H520均购自武汉普诺赛生命科技有限公司，由本实验室保存；Dulbecco’s改良的Eagle’s培养基（Dulbecco’s modified eagle’s medium, DMEM）购自美国Hyclone公司；胎牛血清购自美国Gibco公司；1%青霉素/链霉素、RIPA组织裂解液、BCA蛋白定量试剂盒以及CCK-8试剂盒均购自北京索莱宝生物技术有限公司；Lipofectamine 2000转染试剂购自美国Thermo Fisher公司；过表达lncRNA miR143HG载体、miR-155 mimic及阴性对照均由广州锐博生物科技有限公司提供；TRIzol总RNA提取裂解液购自宝日医生物技术（北京）有限公司；GoTaq®qPCR Master Mix购自普洛麦格（北京）生物技术有限公司；SYBR Green PCR试剂盒购自上海思新生物化学技术有限公司；Wnt抗体、β-Catenin抗体、anti-GAPDH和HRP标记的兔抗山羊IgG均购自美国Abcam公司；低温高速离心机购自美国Thermo Fisher公司；光学显微镜购自日本Olympus公司；多功能酶标仪购自美国BioTek公司；7500型实时荧光定量PCR分析仪购自美国ABI公司；凝胶成像分析仪购自美国Bio-Rad公司。

### 1.2 泛癌及差异分析

在癌症基因组图谱（The Cancer Genome Atlas, TCGA）数据库（https://tcga-data.nci.nih.gov/tcga/）中获取与lncRNA miR143HG相关的多种癌症患者和健康对照的样本数据。使用UALCAN网站（http://ualcan.path.uab.edu）进行lncRNA miR143HG在TCGA数据库的泛癌分析，以反映不同癌症和健康组织中lncRNA miR143HG的表达情况。根据TCGA数据库中LUSC样本和健康对照样本中lncRNA miR143HG的表达量差异，利用秩和检验分析差异显著性。

### 1.3 受试者工作特征（receiver operating characteristic, ROC）曲线和timeROC曲线

在TCGA数据库（https://tcga-data.nci.nih.gov/tcga/）中获取与lncRNA miR143HG相关的502例LUSC患者的样本数据。使用ROC曲线评估lncRNA miR143HG对LUSC患者诊断的预测效果。利用R包‘pROC’分析和绘制ROC曲线并计算曲线下面积（area under the curve, AUC）。使用timeROC评估miR143HG对LUSC患者预后的预测效果。利用R包‘timeROC’分析和绘制1、3、5和10年的timeROC曲线并计算出AUC值。

### 1.4 富集分析

基因集合富集分析（genes set enrichment analysis, GSEA）软件（版本：4.2.3）用于分析各通路对lncRNA miR143HG的富集程度。使用lncRNA miR143HG基因表达量中位数将LUSC样本分成两组（高表达组和低表达组），将LUSC基因表达矩阵输入GSEA软件，选取京都基因与基因组百科全书（Kyoto Encyclopedia of Genes and Genomes, KEGG）中的通路集进行富集分析。

### 1.5 细胞培养

人正常肺上皮细胞BEAS-2B和LUSC细胞H520均使用DMEM培养液（1%青霉素/链霉素+10%胎牛血清）于37 ^o^C和5% CO_2_条件下的恒温恒湿细胞培养箱中培养。根据细胞生长情况每隔2-3 d更换新鲜培养基，进行传代培养。

### 1.6 细胞分组与处理

将人LUSC细胞H520随机分为空白对照组（不做任何处理，blank control，即BC）、阴性对照组（转染lncRNA-negative control，即lncRNA-NC）、miR143HG组（转染lncRNA miR143HG）和miR143HG +miR-155组（共转染lncRNA miR143HG和miR-155）。利用Lipofectamine 2000转染试剂将过表达lncRNA miR143HG的质粒、miR-155 mimic及阴性对照分别转入至H520细胞中，24 h后收集转染细胞进行下一步功能实验。

### 1.7 细胞增殖能力检测

将2×10^3^个转染细胞接种于96孔板中，每孔加入100 μL DMEM完全培养基，孵育24 h。在每孔内均加入CCK-8试剂后避光孵育。2 h后，使用酶标仪测定细胞在450 nm处的吸光度。根据OD_450_计算各组细胞增殖率。细胞增殖率=[（不同时间点的OD_450_均值/0 h OD_450_均值）-1]×100%。

### 1.8 细胞迁移能力检测

将5×10^5^个转染细胞接种于6孔板。培养24 h后，用无菌的200 μ L移液枪头在中间玻片上划痕。分别于0和24 h用显微镜，在100×的放大倍数下记录细胞的迁移情况。并采用ImageJ对0和24 h的图像进行分析，分别计算细胞的迁移率。

### 1.9 细胞侵袭能力检测

将2×10^4^个转染细胞接种于Transwell上室，向下室中加入含10% FBS的培养基600 μL，置于37 ^o^C、5% CO_2_培养箱中。培养24 h后，清洗上室和下室，并在下室中加入400 μL 4%的多聚甲醛，固定10 min。在下室中加入800 μL结晶紫染液染色10 min后，用清水清洗并晾干，于显微镜下观察并拍照。

### 1.10 细胞凋亡率检测

将2×10^6^个转染细胞接种于6孔板。培养24 h后，将细胞消化后收集到离心管内，加入1× Annexin V Binding Solution，制成1×10^6^个/mL的细胞悬液。取100 μL上述细胞悬液，并加入5 μL Annexin V（FITC结合物），再加入5 μL的PI。在室温下避光孵育15 min后，再加入400 μL 1× Annexin V Binding Solution，随后上机检测。

### 1.11 LncRNA miR143HG、miR-155和Wnt/β-Catenin通路相关基因表达检测

收集待测细胞，使用TRIzol裂解液提取总RNA。利用GoTaq®qPCR Master Mix将总RNA逆转录成cDNA。以cDNA为模板进行实时定量聚合酶链反应（quantitative real-time polymerase chain reaction, qRT-PCR）实验，采用2^-ΔΔCt^法计算基因的相对表达量。以GAPDH和U6作为内参基因。引物序列如下所示： lncRNA miR143HG引物上游： 5ʹ-CAGCTCCCAGAACTCGTCCC-3ʹ； lncRNA miR143HG引物下游：5ʹ-CCTCGTCTCCTTTTCCCATGTCT-3ʹ； Wnt引物上游： 5ʹ-CATTGAACAGCTGTGAGCCAT-3ʹ；Wnt引物下游：5ʹ-GGAAATACTGATTCCAGGAGG-3ʹ；β-Catenin引物上游： 5ʹ-TGGCTGTCTAAGCATAGTGATC-3ʹ；β-Catenin引物下游：5ʹ-GGGCAAGATTTCGAATCAATCC-3ʹ； GAPDH 引物上游： 5ʹ-ACAGCAACAGGGTGGTGGAC-3ʹ； GAPDH引物下游：5ʹ-TTTGAGGGTGCAGCGAACTT-3ʹ； miR-155引物上游：5ʹ-CGGCTTAATGCTAATCGTGATAG-3ʹ；miR-155引物下游：5ʹ-GTGCAGGGTCCGAGGT-3ʹ； U6引物上游：5ʹ-CTCGCTTCGGCAGCACA-3ʹ；U6引物下游：5ʹ- AACGCTTCACGAATTT GCGT-3ʹ。

### 1.12 Wnt/β-Catenin通路相关蛋白表达检测

用RIPA组织裂解液冰上裂解细胞。12,000 rpm、4^ o^C离心15 min后收集上清液，使用BCA蛋白定量试剂盒确定总蛋白浓度。每个样品取30 μg总蛋白经10%的SDS-PAGE凝胶电泳分离。电泳结束后，将目的蛋白转移至PVDF膜电转90 min。电转结束后，用TBST稀释的5%的脱脂奶粉室温封闭2 h。随后，将膜与特异性一抗4^ o^C孵育过夜。随后用HRP标记的二抗（1:10,000）室温孵育2 h。PBS洗涤后，用ECL显影液曝光蛋白。ImageJ软件分析蛋白条带的灰度值。各一抗稀释比如下：Wnt（1:1000）、β-Catenin（1:1000）以及GAPDH（1:5000）。

### 1.13 数据分析与处理

使用R语言软件（版本：4.2.1）实现‘pROC’和‘timeROC’分析。在泛癌及差异分析中，两个独立样本的差异分析采用秩和检验。统计数据使用Graph Prism 8.0软件进行分析，符合正态分布的计量资料以均值±标准差表示，采用Student’s t检验进行组间比较。P<0.05为差异具有统计学意义。

## 2 结果

### 2.1 泛癌分析

使用UALCAN网站进行TCGA数据库中lncRNA miR143HG的泛癌分析。结果如图1，在多种癌组织中lncRNA miR143HG的表达明显低于健康组织；而在肾透明细胞癌组织中lncRNA miR143HG的表达明显高于正常组织。

**图1:**
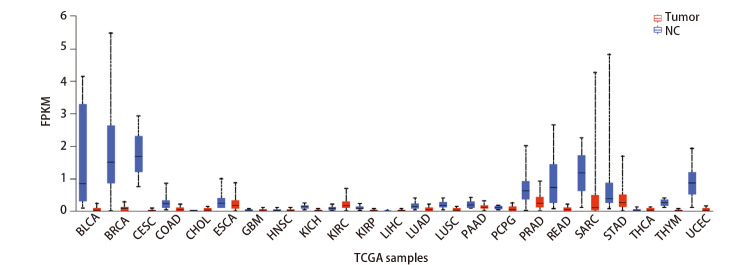
LncRNA miR143HG在各种癌症包括膀胱移行细胞癌、乳腺浸润性癌、宫颈鳞状细胞癌和宫颈内腺癌、结肠癌、胆管癌、食管癌、多形性成胶质细胞瘤、头颈部鳞状细胞癌、肾嫌色细胞癌、肾透明细胞癌、肾乳头状细胞癌、肝脏肝细胞癌、肺腺癌、肺鳞状细胞癌、胰腺癌、嗜铬细胞瘤和副神经节瘤、前列腺腺癌、直肠腺癌、肉瘤、胃腺癌、甲状腺癌、胸腺瘤和子宫内膜癌中的表达情况

### 2.2 差异分析

根据TCGA中LUSC样本和健康对照样本中lncRNA miR143HG的表达量，进行差异表达分析。结果如图2，与健康对照样本比较，LUSC中lncRNA miR143HG表达较低，且差异具有统计学意义（P<0.001）。

**图2:**
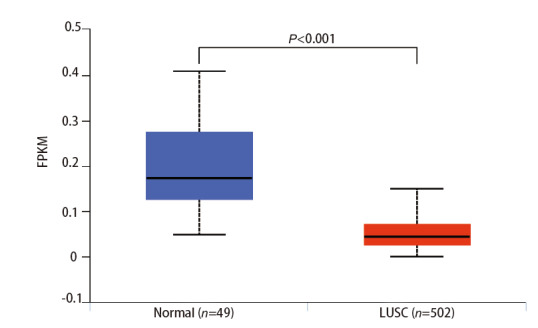
LncRNA miR143HG在正常组织和LUSC中的表达差异（P<0.001）

### 2.3 ROC和timeROC曲线

在TCGA数据库中获取与lncRNA miR143HG相关的502例LUSC患者的样本数据如表1所示。使用ROC曲线评估lncRNA miR143HG对LUSC患者诊断的预测效果。结果如图3A所示，得到的ROC曲线的AUC值为0.926，可认为其对于LUSC具有较高的诊断价值。使用timeROC评估lncRNA miR143HG对LUSC患者预后的预测效果。结果如图3B所示，分别分析和绘制1、3、5和10年的timeROC曲线并计算各自的AUC值，AUC值分别为0.551、0.541、0.546和0.681。由于所得的ROC和timeROC曲线的AUC值均在0.5-1之间，因此lncRNA miR143HG对于LUSC的诊断和预后具有较好的预测效果。

**表1:** TCGA数据库中LUSC患者临床特征统计

Clinical feature	n	Proportion (%)
Age (yr)	492	
≤60	107	22.00
>60	385	78.00
Gender	502	
Male	371	74.00
Female	131	26.00
Smoking status	485	
Smoker	133	27.00
Non-smoker	18	4.00
Reformed smoker	334	69.00
Clinical stage	498	
l	245	49.00
ll	162	33.00
lll	84	17.00
lV	7	1.00

Some data are incomplete or missing. The data presented in the table is the number of patients after filtering out the missing data. TCGA: The Cancer Genome Atlas; LUSC: lung squamous cell carcinoma.

**图3:**
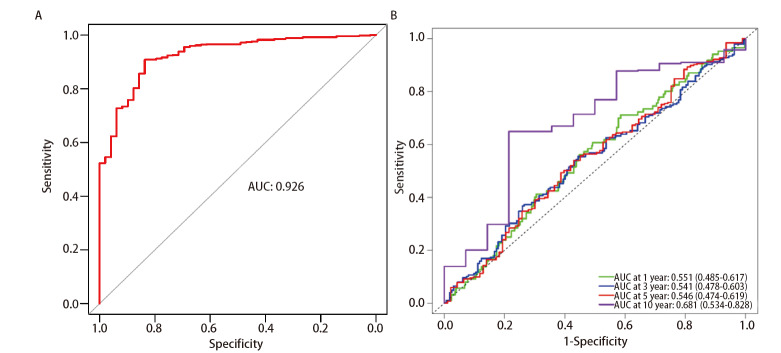
LncRNA miR143HG在LUSC中的诊断和预后的预测效果评估。A：ROC曲线；B：timeROC曲线。

### 2.4 富集分析

通过GSEA和KEGG对lncRNA miR143HG进行富集分析。富集在lncRNA miR143HG高表达组的通路有：黏着斑、血管平滑肌收缩、钙信号通路、细胞黏附因子、转化生长因子-β（transforming growth factor-β, TGF-β）信号通路（表2、图4A）；富集在lncRNA miR143HG低表达组的通路有：氧化磷酸化、细胞周期、基础转录因子、柠檬酸循环、DNA复制等（表2、图4B）。然后将TGF-β信号通路、细胞黏附因子、DNA复制、细胞周期等与肿瘤发生发展密切相关的通路进行可视化，结果如图5所示。

**表2:** 富集在lncRNA miR143HG高表达组和低表达组的通路

Group	Pathway	Size	P
LncRNA miR143HG high expression group	Focal adhesion	199.00	0.001
	Vascular smooth muscle contraction	112.00	0.002
	Calcium signaling pathway	172.00	0.001
	Cell adhesion molecules	130.00	0.004
	TGF-beta signaling pathway	86.00	0.002
LncRNA miR143HG low expression group	Oxidative phosphorylation	131.00	0.010
	Cell cycle	125.00	0.001
	Basal transcription factors	32.00	0.001
	Citrate cycle	30.00	0.006
	DNA replication	36.00	0.001

**图4:**
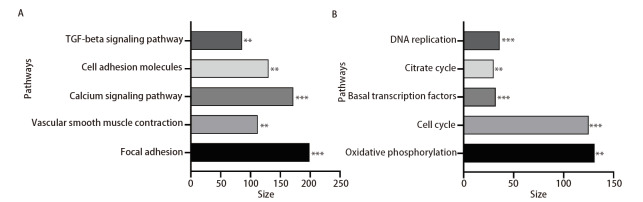
富集在lncRNA miR143HG的通路。A：lncRNA miR143HG高表达组；B：lncRNA miR143HG低表达组。**P<0.01；***P<0.001。

**图5:**
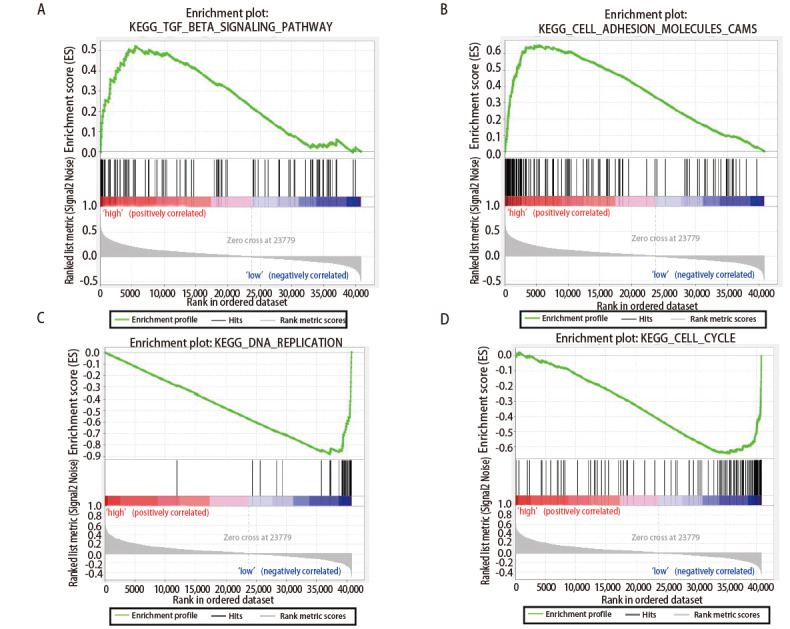
LncRNA miR143HG的部分富集图。A：TGF-β信号通路；B：细胞黏附分子；C：DNA复制；D：细胞周期。

### 2.5 H520细胞和BEAS-2B细胞中lncRNA miR143HG和miR-155表达情况

与人肺正常上皮细胞BEAS-2B比较，lncRNA miR143HG在H520细胞中低表达（1.00±0.04 vs 6.55±0.23, n=3, t=41.18, P<0.001），而miR-155在H520细胞中高表达（1.00±0.09 vs 0.31±0.03, n=3, t=12.60, P<0.001）。另外过表达lncRNA miR143HG后，与NC组比较，发现lncRNA miR143HG在miR143HG组细胞中高表达（2.96±0.26 vs 0.83±0.06, n=3, t=13.83, P<0.001），而miR-155在miR143HG组细胞中低表达（0.21±0.01 vs 1.01±0.04, n=3, t=13.83, P<0.001）。以上结果说明，lncRNA miR143HG在LUSC细胞H520中低表达且负调控miR-155表达。

### 2.6 各组细胞增殖能力

如图6所示，BC组和NC组的细胞增殖能力没有统计学差异（P>0.05）。与NC组比较，转染lncRNA miR143HG后，细胞增殖能力明显降低（P<0.001）；而过表达miRNA-155能促进lncRNA miR143HG介导的细胞增殖能力（P<0.001）。以上结果说明，lncRNA miR143HG能够抑制H520细胞的增殖能力，而过表达miRNA-155能够逆转这一趋势。

**图6:**
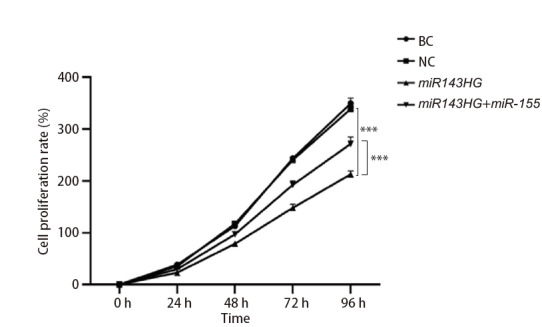
各组细胞增殖能力的比较。***P<0.001。

### 2.7 各组细胞迁移能力

如表3和图7所示，BC组和NC组的细胞迁移能力没有统计学差异（P>0.05）。与NC组比较，转染lncRNA miR143HG后，细胞迁移能力明显降低（P<0.001）；而过表达miRNA-155能够促进lncRNA miR143HG介导的细胞迁移能力（P<0.01）。以上结果说明，lncRNA miR143HG能够抑制H520细胞的迁移，而过表达miRNA-155能够促进lncRNA miR143HG介导的细胞迁移。

**表3:** 各组细胞迁移能力、Wnt蛋白表达以及β-Catenin蛋白表达比较（Mean±SD, n= 3）

Groups	Cell migration rate		Wnt/GAPDH	β-Catenin/GAPDH
	0 h	24 h		
BC	0.00±0.03	0.38±0.05	0.24±0.07	0.25±0.11
NC	0.00±0.04	0.37±0.05	0.27±0.05	0.20±0.06
miR143HG	0.00±0.04	0.18±0.06^***^	1.45±0.09^***^	1.30±0.14^***^
miR143HG+miR-155	0.00±0.02	0.28±0.02^##^	0.85±0.07^###^	0.71±0.01^###^

^**^P<0.001 vs NC group; ^##^P<0.01, ^###^P<0.001 vs miR143HG group; BC: blank control; GAPDH: glyceraldehyde-3-phosphate dehydrogenase.

**图7:**
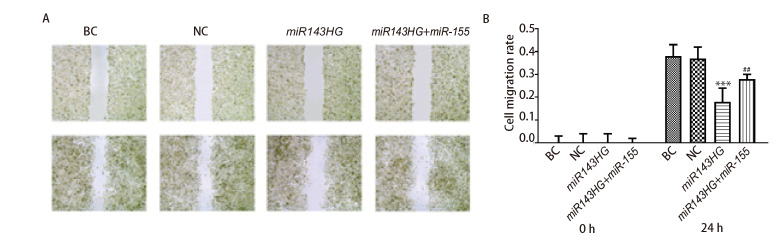
各组细胞迁移能力的比较。A：细胞划痕实验结果；B：各组细胞迁移率。***P<0.001 vs NC组；^##^P<0.01 vs miR143HG组。

### 2.8 各组细胞侵袭能力

如图8所示，与NC组比较，转染lncRNA miR143HG后，细胞侵袭能力明显降低（P<0.001）；而过表达miRNA-155能够促进lncRNA miR143HG介导的细胞侵袭能力（P<0.05）。以上结果说明，lncRNA miR143HG能够抑制H520细胞的侵袭，而过表达miRNA-155能够促进lncRNA miR143HG介导的细胞侵袭。

**图8:**
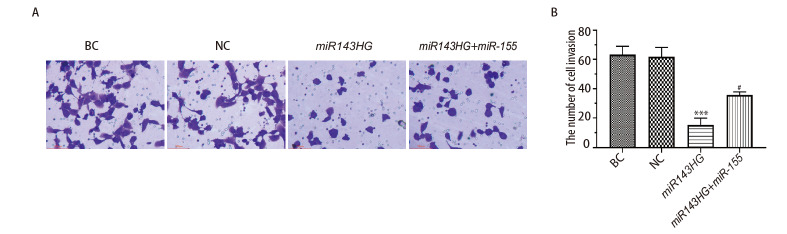
各组细胞侵袭能力的比较。A：结晶紫染色结果（×100）；B：各组细胞侵袭率。***P<0.001 vs NC组；^#^P<0.05 vs miR143HG组。

### 2.9 各组细胞凋亡率

如图9所示，与NC组比较，转染lncRNA miR143HG后，细胞凋亡率明显增加（P<0.001）；而过表达miRNA-155能够抑制lncRNA miR143HG介导的细胞凋亡（P<0.01）。以上结果说明，lncRNA miR143HG能够促进H520细胞凋亡，而过表达miRNA-155能够抑制lncRNA miR143HG介导的细胞凋亡。

**图9:**
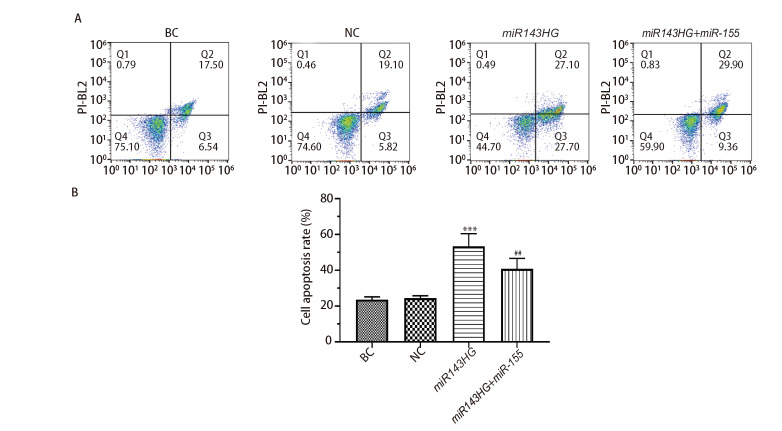
各组细胞凋亡率的比较。A：流式细胞术检测结果；B：各组细胞凋亡率。***P<0.001 vs NC组；^##^P<0.01 vs miR143HG组。

### 2.10 各组细胞Wnt/β-Catenin通路相关基因和蛋白的表达

结果如表2和图10所示，BC组和NC组细胞中Wnt和β-Catenin的基因和蛋白表达没有统计学差异（P>0.05）。与NC组比较，miR143HG组细胞中Wnt和β-Catenin的基因和蛋白表达明显增加（P<0.001）；与miR143HG组比较，miR143HG+miR-155组细胞中Wnt和β-Catenin的基因和蛋白表达明显降低（P<0.01）。以上结果说明，lncRNA miR143HG能够激活Wnt/β-Catenin通路，而过表达miRNA-155能够抑制lncRNA miR143HG介导Wnt/β-Catenin通路活性。

**图10:**
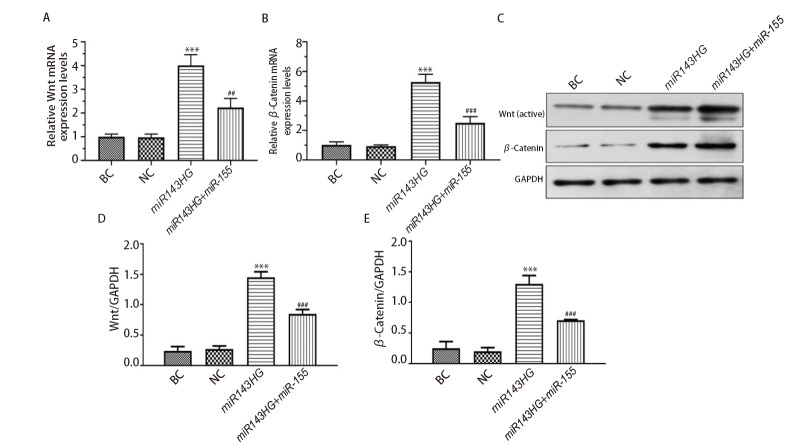
各组细胞Wnt和β-Catenin的基因和蛋白表达水平比较。A：各组细胞Wnt/GAPDH基因表达值比较；B：各组细胞β-Catenin/GAPDH基因表达值比较；C：各组Wnt、β-Catenin和GAPDH的蛋白条带；D：各组细胞Wnt/GAPDH蛋白表达值比较；E：各组细胞β-Catenin/GAPDH蛋白表达值比较。***P<0.001 vs NC组；^##^P<0.01，^###^P<0.001 vs miR143HG组。

## 3 讨论

近年来，lncRNAs受到广泛的关注。多项研究^[24⇓⇓-27]^表明，lncRNAs在多种恶性肿瘤尤其是LUSC的生物学行为中发挥了重要作用。LncRNA miR143HG是一种新的lncRNA，其在肿瘤的生物学行为中的作用研究较少。研究^[28]^发现失调的lncRNA miR143HG与细胞周期阻滞和凋亡密切相关。因此我们首次对lncRNA miR143HG在LUSC中的表达及其发挥的生物学功能进行研究。

本研究发现lncRNA miR143HG在LUSC中低表达，表明lncRNA miR143HG可能在LUSC中发挥着抑癌功能。此外为了进一步揭示lncRNA miR143HG在H520细胞中的生物学功能，本研究通过转染重组质粒来提高H520细胞中lncRNA miR143HG的表达。结果显示，在H520细胞中过表达lncRNA miR143HG能够抑制细胞增殖，降低细胞迁移和侵袭能力，同时促进细胞凋亡。另一方面，qRT-PCR实验发现过表达lncRNA miR143HG能够明显下调miR-155表达，这表明lncRNA miR143HG能够负调控miR-155表达。miR-155作为促癌基因，能够促进细胞增殖，抑制细胞凋亡^[29]^。因此lncRNA miR143HG或许通过下调miR-155表达而发挥其抑癌功能。本研究通过共转染lncRNA miR143HG和miR-155来证实两者的相关性。结果显示，过表达miR-155能够逆转lncRNA miR143HG介导的生物学功能，如细胞增殖和迁移。

高度保守的Wnt信号通路是与癌症密切相关的细胞间分子级联反应^[30]^。值得注意的是，高度保守的Wnt蛋白大小约40 kDa，涉及多种生物学功能，包括干细胞控制、胚胎发生、细胞增殖和转移^[31,32]^。目前已有Wnt信号通路和LUSC的相关研究^[33⇓-35]^，但Wnt通路与lncRNA miR143HG的相关性尚未完全确定。因此本研究进一步探讨lncRNA miR143HG是否影响Wnt信号通路中的蛋白表达。结果显示，过表达lncRNA miR143HG能够激活Wnt/β-Catenin信号通路中相关基因和蛋白表达的活性，这表明lncRNA miR143HG通过调控Wnt/β-Catenin信号通路，从而调控H520细胞的细胞增殖和迁移能力。

综上所述，lncRNA miR143HG在LUSC中低表达且负调控miR-155。且lncRNA miR143HG在功能上通过下调miR-155促进H520细胞的增殖和迁移，其分子机制可能与Wnt/β-Catenin信号通路相关。

**Competing interests**

All authors announce that there are no competing inte trests concerning the pub tlishing of this ar tticle.
